# Asymmetric magnetic proximity effect in a Pd/Co/Pd trilayer system

**DOI:** 10.1038/srep25391

**Published:** 2016-05-06

**Authors:** Dong-Ok Kim, Kyung Mee Song, Yongseong Choi, Byoung-Chul Min, Jae-Sung Kim, Jun Woo Choi, Dong Ryeol Lee

**Affiliations:** 1 Department of Physics, Soongsil University, Seoul 06978, Republic of Korea; 2 Center for Spintronics, Korea Institute of Science and Technology, Seoul 02792, Republic of Korea; 3 Department of Physics, Sookmyung Women’s University, Seoul 04130, Republic of Korea; 4 Advanced Photon Source, Argonne National Laboratory, Argonne, Illinois 60439, USA

## Abstract

In spintronic devices consisting of ferromagnetic/nonmagnetic systems, the ferromagnet-induced magnetic moment in the adjacent nonmagnetic material significantly influences the spin transport properties. In this study, such magnetic proximity effect in a Pd/Co/Pd trilayer system is investigated by x-ray magnetic circular dichroism and x-ray resonant magnetic reflectivity, which enables magnetic characterizations with element and depth resolution. We observe that the total Pd magnetic moments induced at the top Co/Pd interface are significantly larger than the Pd moments at the bottom Pd/Co interface, whereas transmission electron microscopy and reflectivity analysis indicate the two interfaces are nearly identical structurally. Such asymmetry in magnetic proximity effects could be important for understanding spin transport characteristics in ferromagnetic/nonmagnetic systems and its potential application to spin devices.

Spin transport in ferromagnet/nonmagnetic metal (FM/NM) systems have been extensively studied. Interfacial spin-orbit coupling in such systems leads to interesting spin related phenomena and interactions, at the same time providing an effective pathway for electrical manipulation of the magnetization^1^,^2^,^3^,^4^,^5^,^6^,^7^,^8^,^9^,^10^,^11^,^12^,^13^. The spin Hall effect (SHE)^14^,^15^,^16^,^17^,^18^,^19^ in the FM/NM system, in which a charge current in the NM layer produces a spin current into the FM, can be used for reversible magnetization switching of the FM layer^7^,^8^. Current induced spin-orbit torques (SOT) originating from strong interfacial spin-orbit coupling at the FM/NM interface also enables efficient magnetization switching^3^. The interfacial Dzyaloshinskii-Moriya interaction (DMI)^20^,^21^ in FM/NM systems, together with the SHE effect, leads to asymmetric field driven domain wall motion (DWM) and high velocity current-driven DWM^9^,^10^,^22^,^23^,^24^,^25^. In certain FM/NM systems (e.g. Co/Pt, Fe/Pd), the ferromagnet induces a magnetic moment in the adjacent nonmagnetic material due to the magnetic proximity effect^26^,^27^,^28^,^29^,^30^,^31^,^32^,^33^. The magnetic proximity effect must be carefully considered in spin transport measurements since the induced magnetic moment significantly influences spin transport in such systems. Recent studies reveal that the magnetic proximity effect results in modifications to spin transport phenomena such as increased current-driven DWM velocity, reduced SHE, and occurrence of the anomalous Nernst effect^25^,^31^,^32^,^34^,^35^,^36^,^37^,^38^. The magnetic properties of the atoms near the FM/NM interface are important for understanding the interface sensitive magnetic proximity effect. Theoretical and experimental studies also suggest that structural properties of the FM and NM atoms near the interface play a prominent role in determining the spin transport properties^39^,^40^. Therefore, it is important to investigate the structural and magnetic properties at the interface to better understand the spin transport properties of the FM/NM systems.

One interesting aspect of the ferromagnet/nonmagnetic metal systems is the non-equivalent spin transport properties (e.g. SOT, DMI) between NM/FM (FM on top of NM) and FM/NM (NM on top of FM) systems, which has been attributed to the broken spatial inversion symmetry at the FM-NM interface^40^. Even in a symmetric NM/FM/NM structure, the inversion symmetry could be broken if the structural and magnetic properties of the bottom NM/FM and top FM/NM layers are not identical. Earlier studies show that the SOT and DMI do not necessarily cancel out in symmetric NM/FM/NM structures, implying that the structural and/or magnetic properties at the bottom NM/FM and top FM/NM interfaces are distinct. Although the structural difference between the top and bottom interfaces in a NM/FM/NM structure have been observed experimentally, distinguishing possible asymmetry in magnetic properties between the two interfaces has been not trivial. Measuring magnetic properties at the two interfaces separately requires element- and depth-resolved magnetic characterization. In particular, magnetic depth profiles of magnetically induced 4*d* transition metals (e.g. Pd) have been lacking due to the dearth of magnetic scattering beamlines with x-ray energies near the 4*d* transition metal *L* edges (~3 keV). In this study, the depth profiles of the structural and magnetic properties in a NM/FM/NM trilayer system (Pd/Co/Pd) are investigated using x-ray magnetic circular dichroism and x-ray resonant magnetic reflectivity. While the top and bottom interfaces appear to have an almost identical structure, we observe a difference in the induced magnetic moment between the top and bottom NM (Pd). The top NM (Pd) was found to have a significantly thicker “magnetically induced” region and a larger total induced magnetic moment compared to those of the bottom NM (Pd).

## Results

**Figure 1:**
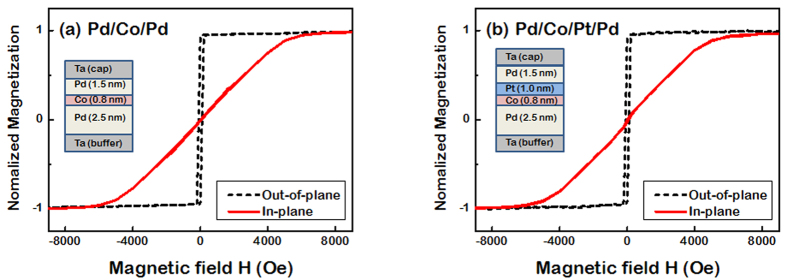
Magnetic hysteresis loops of (**a**) Pd/Co/Pd and (**b**) Pd/Co/Pt/Pd samples measured by VSM. Despite the insertion of the 1 nm thick Pt film between the Co and top Pd, the magnetic hysteresis loops of the two samples are almost identical with a strong perpendicular magnetic anisotropy.

Two separate sets of Pd/Co/Pd thin film structures are selected to study the magnetic proximity effect: (i) Si/SiO_2_/Ta(5)/Pd(2.5)/Co(0.8)/Pd(1.5)/Ta(3) [hereafter referred to as Pd/Co/Pd] and (ii) Si/SiO_2_/Ta(5)/Pd(2.5)/Co(0.8)/Pt(1)/Pd(1.5)/Ta(3) [hereafter referred to as Pd/Co/Pt/Pd]; the number in parenthesis is the nominal film thickness in nm. A 1 nm thick Pt film is inserted between the Co and top Pd in the Pd/Co/Pt/Pd in order to suppress the induced magnetism in the top Pd while not significantly altering the magnetic anisotropy. The magnetic characterization by vibrating sample magnetometry (VSM) shows that both samples have strong perpendicular magnetic anisotropy (Fig. 1). Despite the insertion of a 1 nm thick Pt film between the Co and top Pd in Pd/Co/Pt/Pd, the magnetic hysteresis loops of the two samples are almost identical with out-of-plane magnetic easy axes and large in-plane magnetic saturation fields of ~6 kOe. Both Co-Pd and Co-Pt interfaces are known to have a strong interfacial perpendicular magnetic anisotropy^41^,^42^,^43^,^44^,^45^.

**Figure 2:**
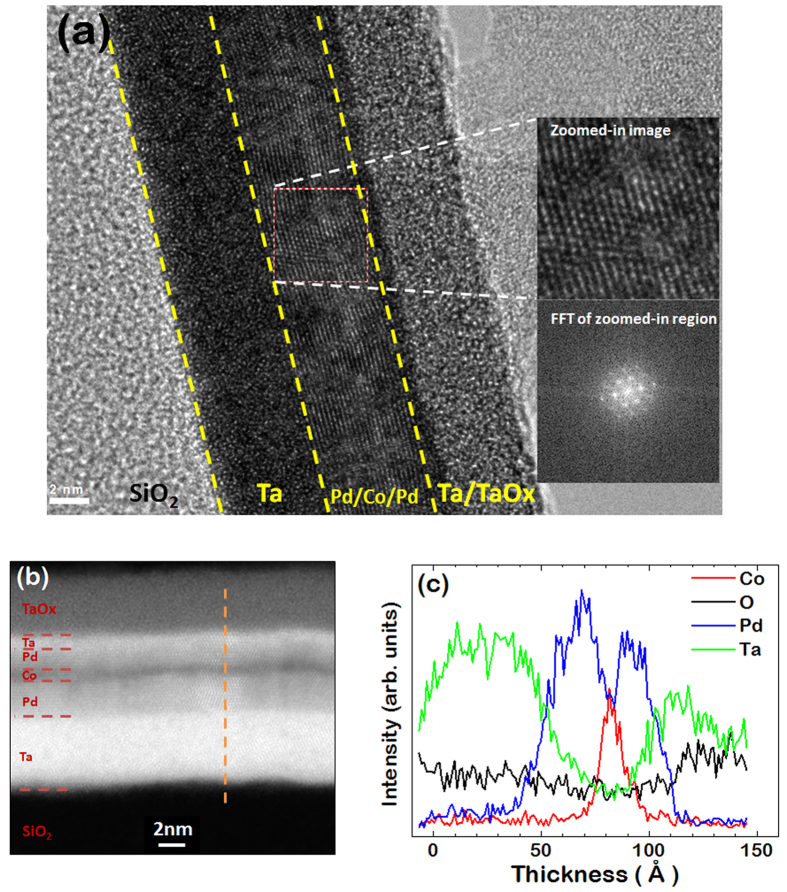
(**a**) TEM image of the Ta/Pd/Co/Pd/Ta film. The zoomed-in image and its FFT show that the Pd/Co/Pd trilayer is epitaxial with an fcc (111) texture. (**b**) STEM image of the Ta/Pd/Co/Pd/Ta film. (**c**) EDX spectra along the orange dotted line in (**b**).

The microstructure of Pd/Co/Pd is investigated by high resolution transmission electron microscopy (HRTEM) and scanning transmission electron microscopy (STEM). The HRTEM image (Fig. 2(a)) reveals that the Pd/Co/Pd film is highly textured on top of an amorphous Ta buffer layer. Although the fast Fourier transform (FFT) indicates that the Pd/Co/Pd has an overall (111) texture, the sporadic dark regions in between the textured region suggest that the Pd/Co/Pd is likely multi-grained. The contrast in the STEM (Fig. 2(b)) is sensitive to the atomic number so that the Co, Pd, and Ta layers are more clearly distinguished. Note that despite a large fraction of the capping Ta layer being oxidized, a thin metallic Ta layer exists between the TaOx and the top Pd that prevents the oxidation of the Pd. Figure 2(c) shows the elemental distribution in the Pd/Co/Pd structure acquired from the energy-dispersive x-ray (EDX) spectrum along the film normal direction. Considerable Ta-Pd and Co-Pd interfacial roughness exists throughout the Ta/Pd/Co/Pd/Ta interfaces. Nevertheless, the Co and Pd films can be clearly distinguished in the STEM image and EDX spectrum.

**Figure 3:**
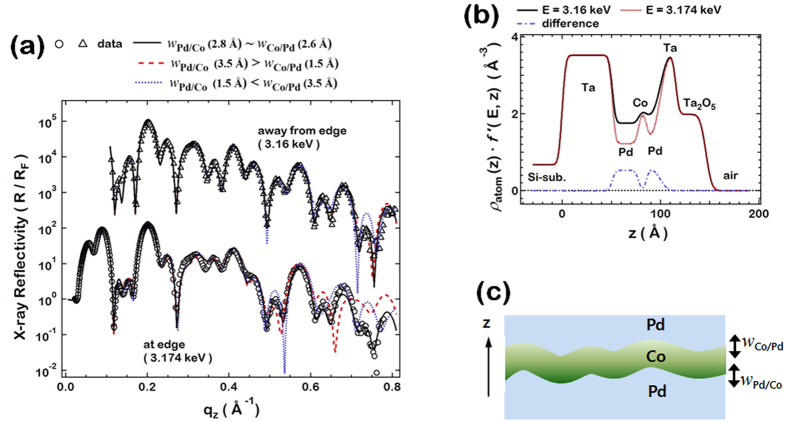
(**a**) X-ray reflectivity intensities measured at the resonant (3.174 keV) and non-resonant (3.16 keV) energies. The reflected intensities were normalized by the Fresnel reflection R_F_ from the ideally smooth Si surface. q_z_ is defined as (4π/λ)sin θ where θ and λ are the angle and wavelength of the incident x-ray. (**b**) Electronic density profiles determined from the best fits for the resonant and non-resonant energies. The difference between the two data is proportional to the density profile of the Pd layers. (**c**) The interfacial roughness at the Pd/Co and Co/Pd interfaces are defined as 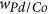 and 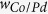, respectively.

In order to clearly quantify and distinguish the structural properties (interfacial roughness, density profile, etc.) of the bottom Pd/Co and top Co/Pd interfaces in Pd/Co/Pd, resonant x-ray reflectivity (RXR) were measured at the Pd *L*_*3*_ edge. RXR is an experimental technique in which the x-ray reflectivity is measured at the absorption edge of a specific element^46^. X-ray reflectivity is widely used to determine the laterally averaged depth profile at the sub-nanometer scale for the total film structure; RXR is an advantageous choice for obtaining a depth profile specific to a certain element, since the atomic scattering factors are dramatically changed around the absorption edge of the specific element of interest. The comparison of the reflectivity intensities measured at the absorption edge and away from the absorption edge can provide an element specific contrast in the total atomic scattering factors, which is then used for obtaining an element specific depth profile (See Supplementary information). The x-ray reflectivity of the Pd/Co/Pd is measured at the Pd *L*_*3*_ edge (E = 3.174 keV) and away from the Pd *L*_*3*_ edge (E = 3.160 keV) as shown in Fig. 3(a). The reflected intensities are normalized by the Fresnel reflection *R*_*F*_ from the ideally smooth Si surface to emphasize the interference patterns due to the film structures. The electronic density profiles are determined from the simultaneous fitting of the two x-ray reflectivity curves in Fig. 3(a). (See Supplementary information) The x-ray reflectivity curves are fitted with multiple parameters such as the film thickness, film density, and interfacial roughness of each layer. The solid lines in Fig. 3(a) represents the calculated x-ray reflectivity based on the best fit model. The interpretation of this data provides a total refractive index profile proportional to the electronic density (Fig. 3(b)), revealing the element-specific depth profile of the elements. Because the x-ray atomic factor of the Pd is the only parameter that could result in the different density profiles between E = 3.174 keV and 3.160 keV, the contrast in the density profiles (dash-dotted blue lines in Fig. 3(b)) corresponds to the element-specific depth profile of the Pd films.

The RXR also provides information about the interfacial roughness in Pd/Co/Pd. The measure of interfacial roughness at the Pd/Co and Co/Pd interfaces are defined as 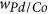 and 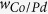, respectively, as depicted in Fig. 3(c). In the best fit model, the interfacial roughness at the bottom Pd/Co interface 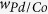 (2.8 Å) and the top Co/Pd interface 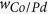 (2.6 Å) are found to be nearly identical. To check the validity of the best fit, reflectivity calculations based on models with different interfacial roughness at the Pd/Co and Co/Pd interfaces are plotted as dashed and dotted lines in Fig. 3(a). The contrast is remarkable at the Pd *L*_*3*_ edge (E = 3.174 keV), where the reflectivity curves of other models (dashed and dotted lines in the bottom panel of Fig. 3(a)) with asymmetric interfacial roughness clearly show deviations from the experimental data. The deviations are prominent at high q_z_’s, whose corresponding length scales are close to the interfacial roughness. Clear deviations between the measured reflectivity and the dashed and dotted lines in Fig. 3(a) demonstrate the high sensitivity of the RXR on the fitting models. Because the RXR curves are best fitted with the structural model with nearly identical (2.6 Å~2.8 Å) interfacial roughness at the top and bottom Pd-Co interface, it is certain that there is little difference in interfacial roughness between the bottom Pd/Co and top Co/Pd interfaces.

**Figure 4:**
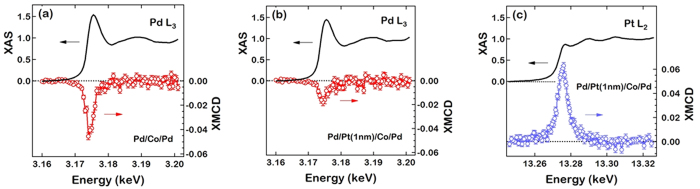
XAS and XMCD measured from the samples Pd/Co/Pd (**a**) and Pd/Pt/Co/Pd (**b,c**). The photon energies of circularly polarized x-rays were tuned to the Pd *L*_*3*_ (**a,b**) and Pt *L*_*2*_ (**c**) edges to extract element-specific information of the induced magnetic moments of Pd and Pt atoms, respectively. The XAS and XMCD intensities were normalized by the averaged XAS intensities over the energies above the absorption edge. It should be noted the overlap of the Ar *K* edge with the Pd *L*_*2*_ edge prevents measurement of the XAS and XMCD intensities at the Pd *L*_*2*_ edge. Similarly the interference between the Ta *L*_*β*_ and Pt *L*_*α*_ fluorescence lines was too strong to measure at the Pt *L*_*3*_ edge.

For element specific magnetic characterizations, the induced magnetic moments of Pd and Pt are measured by fluorescence detected x-ray magnetic circular dichroism (XMCD) with right and left circularly polarized x-rays tuned to the Pd and Pt *L* edges (Fig. 4). XMCD provides direct proof of the magnetic proximity effect by element specific measurement of the induced magnetic moments of nonmagnetic elements in contact with ferromagnetic materials^27^,^28^,^29^,^30^,^32^,^33^. An incident x-ray angle of 3.6 degrees was chosen to maximize the probing volume (due to the larger footprint at grazing angles) and subsequently magnetic sensitivity. The measurements were done with the maximum in-plane magnetic field of ~5 kOe, since XMCD is mostly sensitive to the magnetic moment along the x-ray propagation direction (nearly in-plane at grazing incidence). Although the Pd/Co/Pd and Pd/Co/Pt/Pd films have perpendicular magnetic anisotropy, a large in-plane field rotates the magnetization towards the in-plane direction. The nearly equivalent in-plane magnetic hysteresis loops of the two samples imply that the magnetization directions of the two samples are almost identical with an applied in-plane field of ~5 kOe. The Pd *L*_*2*_ edge could not be measured due to the overlap with the Ar *K* edge of the ambient Ar gas in the x-ray path leading up to the sample. The lack of the XMCD data at the Pd *L*_*2*_ edge prevents us from exactly quantifying the Pd magnetic moment using the XMCD sum rule. The incident x-ray angle of 3.6 degrees ensures that the Pd XMCD measurement probes both of the Pd layers. The path length of the incident x-ray at this angle within any layer (tens of nm) is much smaller than the x-ray absorption length for the layer across the Pd *L*_*3*_ edge (hundreds of nm). Similarly, the total film thickness (<10 nm) is much smaller than the x-ray absorption length for the emitted Pd *L*_*α*_ fluorescence which is at least a few hundreds of nm for any layers. Therefore, contributions from the top and bottom Pd to the measured Pd magnetic signal would be nearly identical if the induced magnetic moments of the two Pd films were equivalent in Pd/Co/Pd.

The XMCD intensities, normalized by the averaged x-ray absorption spectroscopy (XAS) intensities at the energies above the Pd *L*_*3*_ absorption edge, are shown in Fig. 4(a). The peak of the XMCD spectra is −4.7% and −1.9% for Pd/Co/Pd and Pd/Pt/Co/Pd, respectively. The decrease of the XMCD signal in Pd/Pt/Co/Pd compared to Pd/Co/Pd is not unexpected since the 1 nm thick Pt film inserted between the top Pd and Co would reduce the induced magnetic moment of the top Pd. The XMCD peak of Pd/Co/Pt/Pd should be the half of that of Pd/Co/Pd (i.e. −2.35%) in the extreme case that there is no magnetic moment induced in the top Pd in Pd/Co/Pt/Pd due to the inserted 1 nm thick Pt. The magnetic moment of Pd/Co/Pt/Pd (−1.9%) is even less than the half of that of Pd/Co/Pd, implying that the top Pd magnetic moment in the Pd/Co/Pd is larger than the bottom Pd magnetic moment in the Pd/Co/Pd. Pt is an element also known to have the magnetic proximity effect which is confirmed by the XMCD at the Pt *L*_*2*_ edge in Fig. 4(c). In earlier studies the Pt magnetic proximity effect remains up to ~1 nm from the ferromagnetic interface^28^,^29^, so that it is possible that the 1 nm thick Pt film does not completely eliminate the magnetic proximity effect of Pd. If a non-zero magnetic moment exists in the top Pd of the Pd/Co/Pt/Pd, the asymmetry of the induced magnetic moment between the top and bottom Pd would be even larger. From this, it is certain that the top Pd magnetic moment in the Pd/Co/Pd is larger than the bottom Pd magnetic moment.

To confirm the XMCD results that the top Pd has a larger magnetic moment, x-ray resonant magnetic reflectivity (XRMR) of the Pd/Co/Pd was measured. Similar to RXR, which revealed the structural depth profile of the Pd, XRMR analysis can be utilized to determine the depth profile of the induced magnetic moments of Pd atoms. XRMR has been successfully utilized for determination of the laterally averaged depth profile of element specific magnetic moments^47^,^48^ (See Supplementary information). While soft x-ray XRMR has been used to investigate interface magnetism of 3*d* ferromagnetic materials^49^,^50^, intermediate energy x-ray was used in this study to measure the interface magnetism of Pd, a 4*d* metal. For XRMR analysis of the Pd/Co/Pd sample, the scattering intensity of the right (*I*_*+*_) and left (*I*_*−*_) circularly polarized incident x-rays at the Pd *L*_*3*_ edge was measured while applying an in-plane magnetic field of ~5 kOe. It should be noted that in the grazing incidence measurement geometry of the intermediate x-ray XRMR done in our experiments, only the in-plane magnetization would be measureable, since the large angle between the surface normal (perpendicular direction) and the x-ray beam direction would result in small magnetic contrast for perpendicular magnetization. Therefore, all the magnetic x-ray measurements were done with the maximum in-plane magnetic field of ~5 kOe.

**Figure 5:**
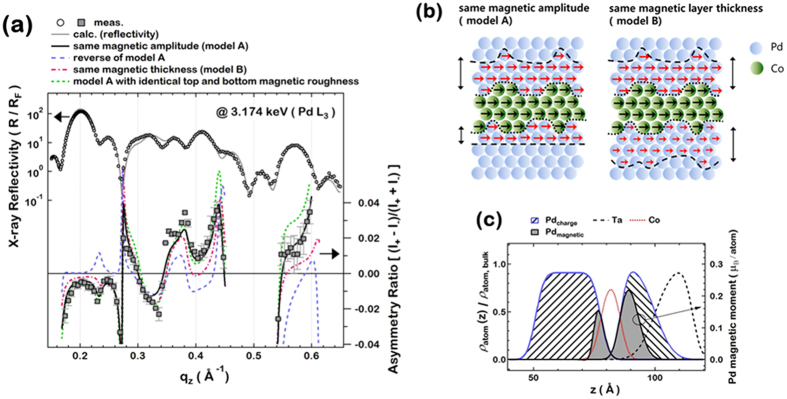
(**a**) Asymmetry ratios (bottom panel), which is defined as (I_+_ − I_−_)/(I_+_ + I_−_) and sensitive to the magnetic moments, and the structure sensitive x-ray reflectivity (top panel) measured at the Pd *L*_*3*_ edge. In the bottom panel, the solid line represents the best fit with model A and the other lines are the calculations with various models. (**b**) Depiction of the magnetic proximity effect of the Pd/Co/Pd system. The top and bottom Pd each have magnetic and nonmagnetic regions. (**c**) Density profiles normalized by the bulk value. The spatial extent of the magnetic region and the total magnetic moment (gray area) is greater for the top Pd. Note that Ta atoms significantly diffuse into the Pd layer on top of the Co layer and overlap the induced Pd magnetic layer.

The Pd magnetic moments is proportional to the asymmetry ratio, 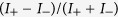, which is defined as the difference in the scattering intensity divided by the sum, shown in the bottom panel of Fig. 5(a). The x-ray reflectivity shown in the top panel in Fig. 5(a) is only sensitive to the structure (electronic density). The asymmetry ratio is fitted with various “magnetic models” to determine the magnetic depth profile. In these models, it is assumed that there are regions of Pd with induced magnetic moments in proximity to Co, and regions of nonmagnetic Pd far away from the Co interface, shown schematically in Fig. 5(b). The “magnetic thickness” of Pd is the spatial extent of the region in which Pd has induced magnetic moments. There also exists a magnetic interface region between the magnetic Pd and its adjacent layers, with an accompanying “magnetic interface roughness”. There are four such interfaces which are depicted as dashed and dotted lines in Fig. 5(b): the two nonmagnetic/magnetic interfaces in the top and bottom Pd, and the two Co-Pd interfaces. We also define the magnetic amplitudes 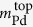 and 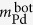, which are the scaling factors for the Pd magnetic moments in the top and bottom Pd magnetic regions, respectively; the estimation of the absolute quantity of the magnetic moment is discussed in the Supplementary information.

In order to understand the asymmetric magnetic moments between the top and bottom Pd layers observed in the XMCD, we assume two magnetic models of the induced Pd magnetic moments: (1) the Pd magnetic layers for the top and bottom Pd layers have identical magnetic amplitudes, 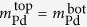, but different magnetic layer thicknesses, 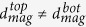 (model A in Figs 5(b) and (2)) identical magnetic layer thicknesses, 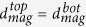, but different magnetic amplitudes, 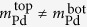 (model B in Fig. 5(b)). The best fit (solid lines in Fig. 5(a)) using model A shows good agreement with the experimental data. In the best fit model, the magnetic amplitude of the magnetic layers is found to be 1.0(±0.1), and the magnetic layer thicknesses are 2.7(±0.2) Å and 6.9(±0.3) Å for the bottom and top Pd layers, respectively. The magnetic interface roughnesses at the purely magnetic interfaces (magnetic Pd-nonmagnetic Pd) are found to be 1.5(±0.2) Å and 3.8(±0.3) Å for the bottom and top Pd layers, respectively. These fitting results indicate that the top and bottom Pd layers have asymmetric magnetic thicknesses and asymmetric magnetic interface roughnesses, with the thicker top Pd magnetic layer having a rougher magnetic interface.

To verify the validity of the asymmetric magnetic roughnesses determined from the best fit, we plot the calculated asymmetry ratios from other models (including model B) in Fig. 5(a), the details of which are discussed in the Supplementary information. The drastic change in the calculated asymmetry ratio with various models clearly shows the sensitivity of XRMR on the magnetic depth profiles; this sensitivity implies that the best fit model may well represent the actual magnetic depth profile. The Pd magnetic depth profile from the best fit model is plotted in Fig. 5(c) overlapped with the elemental electronic density profile normalized by the bulk value. (See Supplementary information) The area of the gray region (Pd_magnetic_) in Fig. 5(c) depicts the total integrated magnetic moment of the Pd atoms. The top Pd layer has a thicker magnetic region (width of the gray region) and larger total integrated magnetic moment (area of the gray region) which explains why the top Pd shows a larger magnetic signal in the XMCD experiments. It is evident that the top and bottom Pd films show asymmetry in the magnetic thickness and total magnetic moment.

## Discussion

We present two possible origins of the asymmetric magnetic proximity effect of Pd observed in this study. First, it should be noted that the top and bottom Pd thicknesses in the Pd/Co/Pd sample, 1.5 nm and 2.5 nm, respectively, are different. Theoretical studies predict a thickness dependent magnetic moment of a Pd film^51^, so that the difference in top and bottom Pd thicknesses could result in different Pd magnetic moments. A systematic Pd thickness dependent study of the Pd magnetic moments in the Pd/Co/Pd structure might be needed in the future to investigate whether the Pd thickness is indeed responsible for the observed asymmetry.

Another possibility on why the top Pd has a larger total magnetic moment than the bottom Pd is the difference in structural properties of the top and bottom Pd layers. It is natural that the Pd film deposited on the Ta buffer (bottom Pd) and the Pd film deposited on the Co film (top Pd) have different structural properties (e.g. lattice constant, strain). Typically, energy band narrowing occurs when the lattice constant increases. Theoretical calculations predict that an increase in lattice constant could result in a magnetic moment in Pd due to an increase in 4*d* DOS near the Fermi level^52^,^53^, while experimental studies show strain induced ferromagnetism in Pd nanoparticles^54^. Then, the thicker top Pd magnetic region in our analysis could indicate that the top Pd has a larger extent of the strained region. Earlier studies on Pd/Co multilayers show that indeed the strained region is larger for the Pd on top of Co^55^. The imperfect crystallinity of our film, evidenced by the dark regions in the HRTEM images in Fig. 2, prevents quantifying structural properties such as the lattice constant or strain. For an in depth structural analysis to distinguish the structure of the top and bottom Pd, a Pd/Co/Pd film with better epitaxial quality, possibly deposited by molecular beam epitaxy (MBE), might be needed. It should be noted that the nearly identical interfacial roughness at the bottom Pd/Co and top Co/Pd interfaces, as discussed earlier with the RXR results, suggests there is no significant difference between the intermixing at the top and bottom Co-Pd interfaces. This eliminates the possibility that a difference in inter-diffusion at the top and bottom Co-Pd interfaces is the origin of the difference in the magnetic proximity effect of Pd.

Any difference in the interfacial structural or magnetic properties greatly influences the spin transport properties in NM/FM/NM systems. For instance, in a Pd/Co/Pd multilayer system, a nonzero spin-orbit field was argued to result from a dissimilar Pd structure on top of and under the Co film^40^. Likewise the different interface structure between the Pt/Co and Co/Pt was used to explain the sizeable difference in the DMI^22^. In addition, the magnetic proximity effect is known to lead to modifications to spin transport phenomena such as increased resistivity, reduced SHE, and increased domain wall velocity^25^,^32^,^34^. Since the magnetic proximity effect is a consequence of the 3*d*–4*d* or 3*d*–5*d* hybridization, it is conceivable that spin transport properties in NM-FM systems, which is sensitive to the interfacial spin orbit coupling, is largely affected by the magnetic proximity effect. Therefore, the asymmetry in the induced magnetic moments should be considered in analysis of the interfacial transport properties. Moreover, the difference in the induced magnetic region of NM/FM and that of FM/NM can be utilized for engineering the spin dependent transport phenomena in spin-orbitronic devices. For example, nonmagnetic materials such as Pd or Pt could be intentionally inserted below or on top of the FM layer in order to enhance or reduce the resistivity, the SHE, the DMI, etc.

In summary, our experiments and analyses provide direct proof that there is indeed significant asymmetry in the interfacial magnetic properties, such as the total induced magnetic moment and magnetic thickness, between the top and bottom nonmagnetic layers in a NM/FM/NM structure, albeit little difference in the interfacial roughness at the two NM-FM interfaces. Further investigation on its exact physical origin, along with its implications on spin transport properties will help us understand the asymmetrical magnetic proximity effect we find in this study.

## Methods

Thin film samples were deposited on a Si/SiO_2_ substrate by dc magnetron sputtering. The base pressure was 1 × 10^−8^ Torr. The microstructure and elemental distribution of Pd/Co/Pd were measured by high resolution transmission electron microscopy (HRTEM), scanning transmission electron microscopy (STEM), and energy-dispersive x-ray (EDX) spectroscopy performed on the cross sectional plane of Pd/Co/Pd using TITAN S 80–300 operated at 300 kV. The x-ray measurements (XMCD, XRMR) were performed at the Advanced Photon Source beamline 4-ID-D. All the experiments were performed at room temperature.

## Additional Information

**How to cite this article**: Kim, D.-O. *et al*. Asymmetric magnetic proximity effect in a Pd/Co/Pd trilayer system. *Sci. Rep*. **6**, 25391; doi: 10.1038/srep25391 (2016).

## Supplementary Material

Supplementary Information
