# Supplementary material for: Asymmetric magnetic proximity effect in a Pd/Co/Pd trilayer system
Source: Sci Rep. 2016 May 6;6:25391. doi: 10.1038/srep25391 (PMC4858727; doi:10.1038/srep25391)
Supplement: Supplementary Information [file srep25391-s1.pdf]

Supplementary information

Asymmetric magnetic proximity effect in a Pd/Co/Pd trilayer system

Dong-Ok Kim[1,2], Kyung Mee Song[2,3], Yongseong Choi[4], Byoung-Chul Min[2], Jae-Sung Kim[3], Jun Woo Choi[2,a)], Dong Ryeol Lee[1,b)]

[1]*Department of Physics, Soongsil University, Seoul 06978, Republic of Korea*

[2]*Center for Spintronics, Korea Institute of Science and Technology, Seoul 02792, Republic of Korea*

[3]*Department of Physics, Sookmyung Women's University, Seoul 04130, Republic of Korea*

[4]*Advanced Photon Source, Argonne National Laboratory, Argonne, Illinois 60439, USA*

a) junwoo@kist.re.kr

b) drlee@ssu.ac.kr

Resonant x-ray reflectivity

X-ray reflectivity is sensitive to the gradient of the x-ray refractive index along the surface normal direction (z direction) which is used to determine the laterally averaged depth profile. The x-ray refractive index *n(z,E)* is given by

$$n(z,E) = 1 - \frac{\lambda^2 r_e}{2\pi} \sum_i \rho_{\text{atom},i}(z)\left[f_i'(E) + i f_i''(E)\right] \qquad (1)$$

where λ is x-ray wavelength, r_e is the classical electron radius, the summation is over constituent atoms, and ρ_{atom} is the atom number per unit volume (atomic density). $f' + i f''$ is the complex atomic scattering factor, whose imaginary part is related to x-ray absorption, and the real part is related to the imaginary part by the Kramers-Kronig dispersion relation. The x-ray reflectivity intensities measured at grazing incidence angles can be calculated explicitly using the Parratt's recursive formalism[1] , which is an extension of optics theory to x-rays, with additional modification assuming a Gaussian distributed interfacial roughness. A Debye-Waller factor like form derived in the distorted wave Born approximation (DWBA) has been widely used[2].

Figure S1 shows the atomic scattering factors of the Pd atoms in Pd/Co/Pd near the Pd L_3 edge. The imaginary scattering factor f'' was obtained from the measured XAS data (Fig. 4(a)), which was then transformed into the real part f' using the Kramers-Kronig relation[3]. Both procedures use tabulated factors from away from the absorption edge for scaling (normalization)[4]. Since other constituent elements' scattering factors are almost constant, any change in reflectivity intensities at and away from the Pd L_3 edge results from the contrast in refractive indices at the Pd-Co and Pd-Ta interfaces. The vertical bars (blue) in Fig. S1 indicates that the contrast between the atomic scattering factors of Pd and Co atoms is

remarkably enhanced at the Pd L_3 edge (E = 3.174 keV) when compared with those away from the edge (E = 3.160 keV). This energy dependent contrast results in a clear difference between the x-ray reflectivity curves measured at the two energies near the Pd L_3 edge, as shown in Fig. 3(a).

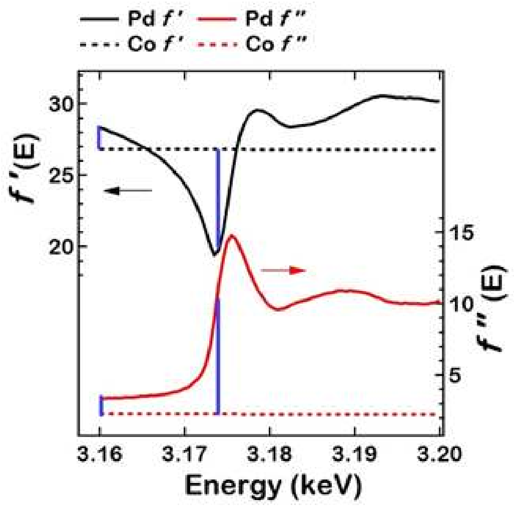

Figure S1. Atomic factors of the Pd atoms in Pd/Co/Pd near the Pd L_3 edge.

X-ray reflectivity fitting

For simultaneous fitting of the two x-ray reflectivity curves in Fig. 3(a), an identical set of structural parameters are used. This is due to the fact that only a noticeable difference in the physical parameters describing the two reflectivity curves at the different x-ray energies is the Pd atomic scattering factors. Considering the Gaussian distributed interface profiles assumed in the DWBA, the total refractive index profile can be calculated using an error function, the derivative of which is a Gaussian function. Figure 3(b) shows the total refractive index profiles, explicitly $\rho_{atom}(z) \cdot f'(\mathrm{E}, \mathrm{z})$, for the two different energies determined

from the best fit structure. Since the difference between two profiles (dash-dotted blue lines) only results from different atomic factors of Pd layers, it determines an element-specific depth profile of the Pd layers, which corresponds to

$$\rho_{\mathrm{atom,Pd}}(z)\cdot\left[f'_{\mathrm{Pd}}(3.16\,\mathrm{keV}) - f'_{\mathrm{Pd}}(3.174\,\mathrm{keV})\right]$$

X-ray resonant magnetic reflectivity

Similar to conventional x-reflectivity, x-ray resonant magnetic reflectivity is sensitive to the gradient of x-ray refractive index originating from the magnetic response. Typically the magnetic response in the x-ray refractive index is too small to be measurable, but it can be enhanced and observable when the incident x-rays are circularly polarized and the x-ray energy is tuned to the absorption edge of a specific magnetic element. Therefore, XRMR can determine laterally averaged depth profile of element-specific magnetic moments. Following Ref. 5, the magnetically sensitive part component is added to the complex x-ray atomic scattering factors for the magnetic element atoms. Then the atomic scattering factor in eq. (1) is given as

$$f'_{tot}(E)+i\,f''_{tot}(E)=\left(\hat{e}_f^{*}\cdot\hat{e}_i\right)\left[f'_c(E)+i\,f''_c(E)\right]+i\left(\hat{e}_f^{*}\times\hat{e}_i\right)\cdot\hat{m}(z)\left[f'_m(E)+i\,f''_m(E)\right] \qquad (2)$$

where $\hat{e}_{i(f)}$ and $\hat{m}$ are unit vectors of the incident(outgoing) x-ray polarization and magnetization of the magnetic layers, respectively. The magnetic atomic factors f'_m and factors f''_m are only detectable near the absorption edges of magnetic elements. Figure S2 shows the Pd magnetic atomic factor $f''_m(E)$ obtained from the measured XMCD in Fig. 4(a) with the same scaling factor used for the charge atomic factor $f''(E)$. The real part f'_m was obtained using the Kramers-Kronig relation.

Since the magnetic atomic factors are typically obtained from XMCD results, a homogeneous magnetization is assumed inside the magnetic layer, since XMCD is a bulk sensitive measurement. However, since Pd atoms are only magnetized near the Pd-Co interfaces, we added a factor $r(z)$ with a relative spatial distribution to the magnetic atomic factors as $i\left(\hat{e}_f^* \times \hat{e}_i\right)\cdot \hat{x}\, r(z)\left[f_m'(E) + i\, f_m''(E)\right]$. Since only the saturation magnetization is of interest, it is assumed that $\hat{m}(z) = \hat{x}$, which is along the x-ray beam direction in the sample surface plane.

To consider explicitly the polarization dependence of magnetic atomic factors in eq. (2), a full dynamical formalism or optical formula extended for polarized x-rays and resonant scattering amplitudes should be used[6]. However, a more realistic model with both structurally and magnetically rough interfaces can be taken into account in the DWBA formula. The magnetic DWBA formula can be simplified for circularly polarized x-rays in an approximation of grazing incidence angles, relatively weak resonant scattering amplitudes, and dominant magnetization along the beam direction in the sample surface plane[7]. Since our experimental conditions meet this approximation, we have used the simplified DWBA formula to explain quantitatively the magnetic depth profile of induced Pd magnetic moments. As in RXR, we assume a Gaussian distributed interfacial roughness in the DWBA formula used for the XRMR analysis to obtain the relative magnetic depth profile shown as the shaded area in Fig. 5(c).

.

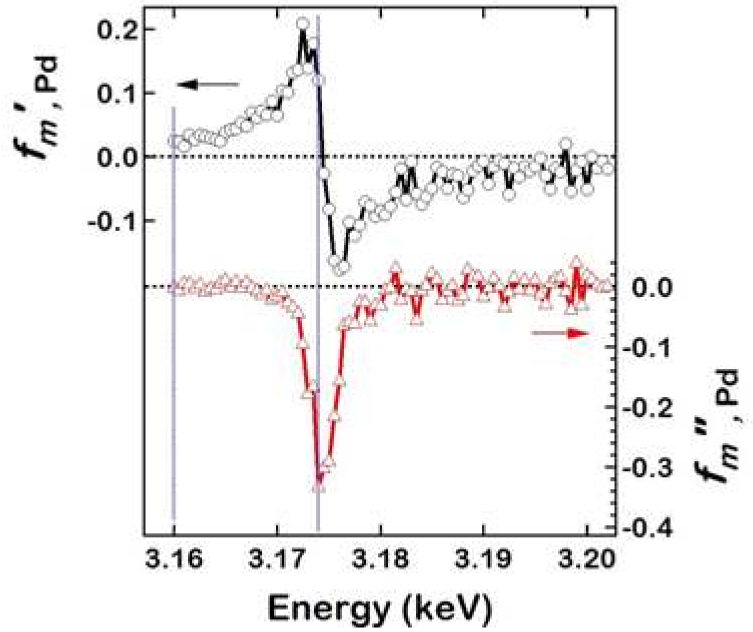

Figure S2. Magnetic atomic factors of the Pd atoms in Pd/Co/Pd near the Pd L_3 edge.

X-ray resonant magnetic reflectivity fitting with various models

To verify the validity of the asymmetric magnetic roughnesses determined from the best fit, we plot the calculated asymmetry ratios (green dotted lines in Fig. 5(a)) with identical magnetic roughness (1.5Å) at the top and bottom nonmagnetic Pd-magnetic Pd interface and other parameters unchanged. The green dotted line shows a clear deviation from the experimental data at high q_z's. Additionally, the calculated asymmetry ratio for the reverse case in which the parameters of the magnetic layers used for the best fit are switched for the top and bottom Pd layers ($d_{mag}^{top} < d_{mag}^{bot}$), is shown as the dashed blue lines in Fig. 5(b). The deviation from the measured data is obvious, in particular the reversed sign of the asymmetry ratio near $q_z \sim 0.23$ Å^{-1}. Finally, the calculated asymmetry ratio based on the best fit using model B (symmetric magnetic thicknesses but asymmetric magnetic amplitude) are shown as

the red dash-dotted lines in Fig. 5(a). The deviation from the experimental data is clear at high q_z's. It should be noted that this deviation is not improved by varying the fitting parameters. Since the magnetic moments of Pd induced by the Co atoms should result from the Pd 4*d*-Co 3*d* hybridization, the nearly identical chemical (electronic) structures at the Pd/Co and Co/Pd interfaces from the RXR analysis (Fig. 3) suggest that the induced magnetic moment per Pd atom should be similar between the top and bottom Pd films. Then it is plausible that, model A, with identical magnetic amplitudes $m_{\mathrm{Pd}}^{\mathrm{top}} = m_{\mathrm{Pd}}^{\mathrm{bot}}$ but different magnetic layer thicknesses $d_{mag}^{top} \neq d_{mag}^{bot}$, is more valid for explaining the measured asymmetry ratio.

Estimation of Pd magnetic moment

The lack of XMCD data at Pd L_2 edge prevents us from quantifying the Pd induced magnetic moment using the sum rule. According to Bailey *et. al.* , it can be estimated that a maximum Pd XMCD signal of -10 % (normalized by the step height at the Pd L_3 edge) correspond to a total magnetic moment of 0.116μ_B/atom[8]. Then a maximum XMCD signal of -4.7 %, as shown in Fig. 4(a), would correspond to 0.05μ_B/atom. The difference in the Pd moments is possibly due to the fact that there is some inaccuracy in the Pd magnetic moment values deduced from XMCD data in earlier studies, since the exact magnetic thickness is unclear. To estimate the absolute value of induced Pd magnetic moments, the following relationship between the XMCD and XRMR results should be considered:

$$m_{\mathrm{tot,Pd}} = \overline{m}_{\mathrm{Pd,XMCD}} \int \rho_{\mathrm{atom,Pd}}(z)\, dz = \overline{m}_{\mathrm{Pd,XRMR}} \int \rho_{\mathrm{atom,Pd}}(z)\, r(z)\, dz \qquad (3)$$

where $\overline{m}_{\mathrm{Pd,XMCD}}$ and $\overline{m}_{\mathrm{Pd,XRMR}}$ are the average magnetic moments for the XMCD and

XRMR data, respectively. The magnetic depth profile r(z) was determined from the best fit using a model where each induced magnetic layer has same magnetic moment within the layer and different magnetically rough interfaces. From Eq. (3), the corrected magnitude of the Pd magnetic moments, $\overline{m}_{\mathrm{Pd,XRMR}}$, is estimated to be 0.3$\mu_B$/atom. This magnitude is consistent with the result of Vogel *et. al.* for a 0.7 nm thick Pd in between Fe layers, where the Pd layers were assumed to be fully magnetized[9]. It should also be noted that the calculated induced Pd magnetic moment in Fe/Pd(001) was found to be 0.36~0.38μ_B/atom within two layers from the Fe-Pd interface[10]. The distribution of the induced Pd magnetic moments in Fig. 5(c) is plotted in units of μ_B/atom calculated by $\overline{m}_{\mathrm{Pd,XRMR}} \cdot r(z)$.

REFERENCES

1. Parratt, L. G. Surface studies of solids by total reflection of X-rays. *Phys. Rev.* **95**, 359 (1954).

2. Sinha, S. K., Sirota, E. B., Garoff, S. & Stanley, H. B. X-ray and neutron scattering from rough surfaces. *Phys. Rev. B* **38**, 2297 (1988).

3. Cross, J. O. *et al*. Inclusion of local structure effects in theoretical x-ray resonant scattering amplitudes using *ab initio* x-ray absorption spectra calculations. *Phys. Rev. B* **58**, 11215 (1998).

4. Henke, B. L., Gulikson, E. M. & Davis, J. C. X-ray interaction: Photoabsorption, scattering, transmission, and reflection at E=50-30,000 eV, Z=1-92. *At. Data Nucl. Data Tables* **54**, 181 (1993).

5. Hannon, J. P., Trammell, G. T., Blume, M. & Gibbs, D. X-ray resonance exchange scattering. *Phys. Rev. Lett.* **61**, 1245 (1988).

6. Stepanov, S. A. & Sinha, S. K. X-ray resonant reflection from magnetic multilayers: Recursion matrix algorithm. *Phys. Rev. B* **61**, 15302 (2000).

7. Lee, D. R. *et al*. X-ray resonant magnetic scattering from structurally and magnetically rough interfaces in multilayered systems. I. Specular reflectivity. *Phys. Rev. B* **68**, 224409 (2003).

8. Bailey, W. E. *et al*. Pd magnetism induced by indirect interlayer exchange coupling. *Phys. Rev. B* **86**, 144403 (2012).

9. Vogel, J. *et al*. Structure and magnetism of Pd in Pd/Fe multilayers studied by x-ray magnetic circular dichroism at the Pd $L_{2,3}$ sedges. *Phys. Rev. B* **55**, 3663 (1997).

10. Lee, S.-K. *et al*. Surface alloying and magnetism of ultrathin Fe films on Pd (001). *Phys. Rev. B* **65**, 014423 (2001).
